# Adrenal Cortical Rests in the Fallopian Tube: Report of a Case and Review of the Literature

**DOI:** 10.3390/medicines8030014

**Published:** 2021-03-12

**Authors:** Theodoros Tzigkalidis, Eleni Skandalou, Maria Eleni Manthou, Nikolaos Kolovogiannis, Soultana Meditskou

**Affiliations:** 1 Pathology Department, General Hospital “Agios Pavlos”, 55134 Thessaloniki, Greece; theo.tzig.80@gmail.com; 2 Internal Medicine Department, General Hospital “Papanikolaou”, 57010 Thessaloniki, Greece; skandaloueleni@gmail.com; 3 Laboratory of Histology-Embryology, School of Medicine, Faculty of Health Sciences, Aristotle University of Thessaloniki, 54124 Thessaloniki, Greece; meditskou@gmail.com; 4 Obstetrics and Gynecologic Department, General Hospital of Polygyros, 63100 Chalkidiki, Greece; kolnik2011@hotmail.com

**Keywords:** adrenocortical rest, ectopic adrenal, fallopian tube, adrenal ectopy, adult

## Abstract

We report an extremely unusual finding discovered incidentally during a routine pathologic evaluation of a fallopian tube, surgically removed on the grounds of ectopic pregnancy. We came across a minute yellowish nodule situated within the wall of the salpinx, which corresponded to ectopic adrenal cortex, as verified by microscopical examination, and coexisted with salpingitis isthmica nodosa. A research of the available English literature on this subject confirmed the rareness of the entity we encountered. This case is presented because of its uniqueness, aiming to raise awareness of a rare condition which is discussed along with potential diagnostic dilemmas, its prognostic significance, and possible complications.

## 1. Introduction

Adrenal glands are normally situated on the kidneys and have a double embryological origin [1]. Ectopias of the adrenal gland, and especially of its cortex, are mostly reported during childhood and may be encountered in association with pelvic structures, usually those of the genitourinary system, and usually in males [2,3]. Adrenal cortical rests occur in 50 per cent of new-borns and usually regress and disappear within a few years [3], but they may remain and persist as functioning tissue throughout adulthood [1]. Εctopic adrenal tissue is generally rarely observed and is usually a random finding [2].

We report an extremely unusual finding of adrenal cortical rest within the wall of salpinx, which is an extremely rare site [4,5], in an older female, which is even more unusual. The lesion was discovered incidentally during a routine pathologic evaluation of a fallopian tube, surgically removed on the grounds of ectopic pregnancy. This case is presented because of its uniqueness, aiming to raise awareness of a rare condition which is discussed along with the pathogenetic mechanism responsible for the lesion’s occurrence, potential diagnostic dilemmas, its prognostic significance and possible complications.

## 2. Case Presentation

A 37-year-old female patient visited the local hospital unit complaining of pelvic pain and discomfort, combined with vaginal bleeding. She reported that symptoms began a few days earlier and that her last menstruation was documented 7 weeks prior to the symptoms. Her personal medical history only included a surgically removed right ovarian cyst 5 years before, which was diagnosed as a benign serous cystadenoma.

A blood count was performed, revealing normal values. CEA (carcinoembryonic antigen), aFP (alpha fetoprotein), CA19-9 (carbohydrate antigen 19-9) values were within normal ranges. Serum CA-125 (cancer antigen 125) was found at 18.6 IU/mL (the upper normal value is 35 IU/mL). βHCG (beta human chorionic gonadotropin) was measured at 11,564 mIU/mL, suggesting pregnancy. An endometrial curettage was performed and βHCG dropped to 9541.92 mIU/mL. Thorough ultrasonographic examination was performed, which indicated an ectopic pregnancy in the right fallopian tube. It was therefore immediately decided to have it surgically removed. After surgery, βHCG dropped to 2418.74 mIU/mL. All specimens were sent to the laboratory for histological evaluation and diagnosis.

Macroscopically, the salpinx measured 8 × 2.5 cm and appeared intact but was obviously dilated in the middle of its ampullary region. The lumen seemed to be filled with clots of blood. The organ was cut perpendicularly to its long axis at sequential sections, measuring approximately 0.5 cm each. During sectioning, a minute, yellowish and firm nodule was found located within the wall of the salpinx, measuring no more than 2 mm. It was prepared for microscopic investigation.

**Figure 1:**
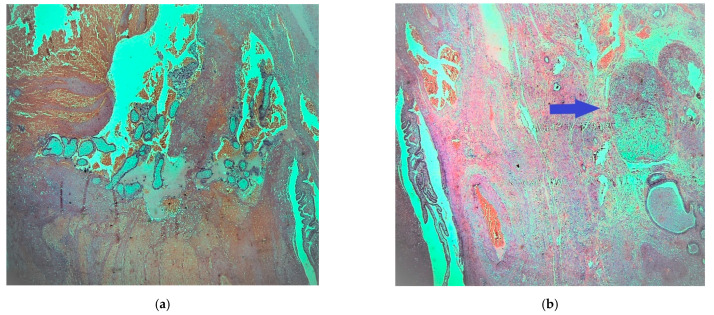
Fallopian tube: (**a**) fibrotic chorionic villi and trophoblast in the lumen of the salpinx (H & E, ×40); (**b**) demarcated nodule in the fallopian fibromuscular layer (blue arrow) (H & E, ×20).

Microscopically with H&E (hematoxylin eosin) staining, many chorionic villi and trophoblastic cell islands were observed between fibrin thrombi and the luminal blood clots in the salpinx (Figure 1a). The endometrial samplings exhibited morphological features of a hypersecretory or gestational endometrium, obviously caused by increased progesterone effect, confirming the initial clinical diagnosis of ectopic tubal pregnancy. Meanwhile, the very small nodule discovered was located between bundles of smooth muscle cells of the outer longitudinal muscularis layer, with interspersed loose connective tissue of serosa present. The nodule appeared well demarcated, it was surrounded by a thin fibrous rim and was composed of two intermingled but distinct cell populations, arranged in small parallel cords and trabeculae (Figure 1b).

**Figure 2:**
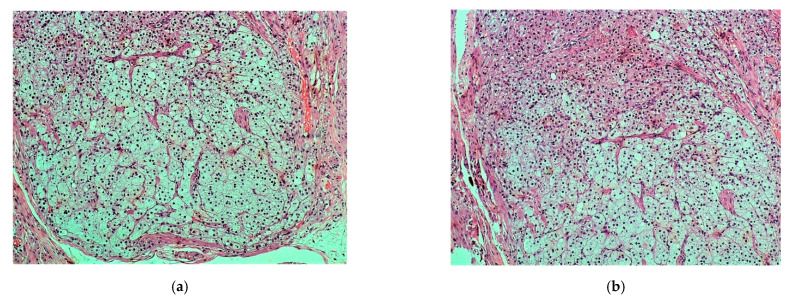
(**a**,**b**). Nodule: vacuolated lipid-rich cell population (zona fasciculata) admixed with a lesser population of eosinophilic or amphophilic cells (zona glomerulosa) (H & E, ×100).

All cells were clearly outlined, with small, uniform and pycnotic nuclei, which had no signs of atypia or mitoses. Many cells appeared with clear and abundant, microvesicular (lipid-rich) cytoplasm, while some had amphophilic to slightly eosinophilic cytoplasm (Figure 2).

**Figure 3:**
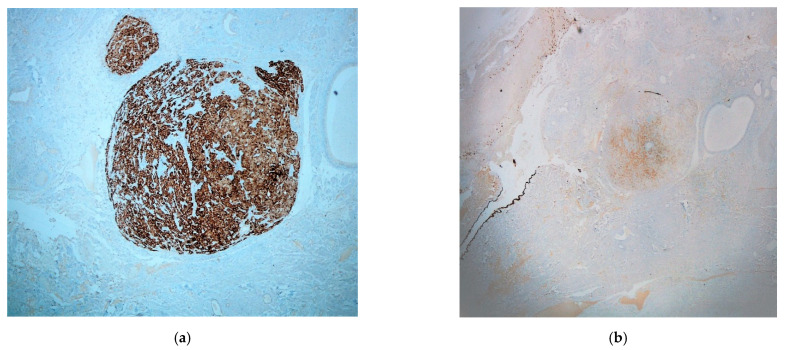
Immunohistochemistry: (**a**) Melan-A (MART1) (×40); (**b**) calretinin (×20) (mesothelial cells of the fallopian serosa serve as the internal positive control).

Preliminary differential diagnosis included a variety of entities, such as Walthard cell nests, accumulation of foamy histiocytes, metastatic renal clear cell carcinoma, displaced ovarian luteinized theca cells, heterotopia of ovarian hilus cells and, lastly, ectopic adrenal cortex. Immunohistochemical evaluation was performed by a two-step Biotin complex EnVision™+ System (Dako Cytomation, Carpinteria, CA, USA). It revealed a widespread, strong positivity of the described cell population for Melan-A (MART1) (Figure 3a) and an extensive, but less intense positivity for calretinin (Figure 3b), a-inhibin and synaptophysin. Cytokeratins AE1/AE3, CD10, S-100 protein, HMB45 and chromogranin, were all negative. The results confirmed the existence of adrenocortical rest in the fallopian tube. Within muscular and serosa layers, cystically dilated glands were also observed, lined by ciliated columnar epithelium, without atypia. The glands were surrounded by smooth muscle or dense fibrous tissue, features indicating the presence of salpingitis isthmica nodosa.

## 3. Discussion

The presence of adrenal remnants away from the normally expected site of glands has been documented a long time ago and was first described by Morgagni himself in the 18th century [1]. Adrenal remnants are often discovered in the genitourinary system during early childhood in both sexes, more frequently in males, most of them being located in the groin region [2,3,6,7,8]. Other reported settlement areas for ectopic adrenal rests are the kidney, liver, pancreas, colon, celiac plexus, placenta, ovary and retroperitoneal area [5]. Adrenal remnants in females is a very rare situation, but if they do occur, the sites of predilection are the broad ligaments [4]. It is reported that almost 25% of the excised broad ligaments, provided they are completely and carefully examined, may reveal remnants of adrenocortical tissue [9,10].

These remnants usually consist exclusively of cortical cells, lacking any medullary cells of the normal adrenal, which can be explained by the different embryological origin of the two components [3,11]. There are two different primordia of separate origin: the cortex is derived from the mesoderm and the medulla from chromaffin neuroectodermal cells of the neural crest [1]. During embryonic development they merge into a single unit [3]. Over the course of this time small fragments of the cortex can be entrapped in the descending gonads and engulfed in the developing organs [11,12]. These remnants most of the time become atrophic and disappear until adolescence, because the normal functioning adrenal glands decrease their hormonal stimulation [3,12]. However, these structures may exceptionally escape this rule and acquire hyperplastic, hyperfunctioning or even neoplastic potential [5,12,13,14,15,16,17,18]. Occasionally, microscopic ectopic rests may persist and may be discovered incidentally in organs or at the periphery of coexisting tumors removed surgically from patients. As far as we know, this is only the second reported case until now in the English literature, documenting adrenal rests in the fallopian tube, confirming the rarity of the lesion at this specific site [4]. In addition, it is the first time it is found combined with salpingitis isthmica nodosa, which may explain the ectopic pregnancy [19,20].

The diagnosis is straightforward on most occasions, based on cytological, architectural and, when necessary, immunohistochemical characteristics.

Traditionally, adrenal cortical cells reveal immunohistochemical positivity to Melan A, inhibin, synaptophysin [6,21,22,23], calretinin [22,23] and also exhibit high nuclear positivity (86%) to SF-1 (antisteroidogenic factor-1) [22]. Ovarian hilus cell heterotopia of the fallopian tube shows similar immunopositivity, while renal clear cell carcinoma is negative to all of the above markers. Amongst these markers in our case, SF-1 was not performed, because it is not a routine available marker in our laboratory.

Adrenal cortical cells are negative for AE1/AE3, CD10, which are usually positive markers for renal clear cell carcinoma [6,21,22,23]. The cells are also negative for chromogranin, a positive marker in hilus cell heterotopia [21,22,23]. It should be pointed out that hilus cell heterotopia is an undoubtedly unusual but benign finding, having many immunohistochemical similarities with adrenal cortical rests, although both cytological and architectural features differ [24].

The clinical significance of adrenal remnants is usually not critical and is commonly not related with endocrine irregularities [5]. Nevertheless, it is important to realize that ectopic tissue may develop the same pathologies as the normal adrenal gland [25]. Theoretical implications include secondary hyperplasia occurring after adrenalectomy, adrenal insufficiency in certain patients, and the possibility of neoplastic transformation [2,3].

## 4. Conclusions

Ectopic adrenocortical rests in the adult population constitute a rare entity, which is less frequent in women and even more unusual when located in fallopian tubes. It usually has limited clinical significance because it is generally asymptomatic, and it is often discovered incidentally after surgery. Despite its indolent course, it would be wise to have it excised whenever encountered during surgery, because it can potentially become functional or even malignant [12,26]. Awareness from the side of both surgeons and pathologists about the existence of ectopic adrenal tissue is critical to avoid misinterpretation. Such unexpected findings can offer an insight into the complex and sometimes unpredictable events that may take place during embryogenesis, a fact that should be considered in every histological specimen that is examined, even those that seem scientifically bland or uninteresting.

## Data Availability

Data available on request due to ethical restrictions. The data presented in this study are available on request from the corresponding author. The data are not publicly available because of personal data protection restrictions.
